# Antidepressants and Violence: Problems at the Interface of Medicine and Law

**DOI:** 10.1371/journal.pmed.0030372

**Published:** 2006-09-12

**Authors:** David Healy, Andrew Herxheimer, David B Menkes

## Abstract

David Healy and colleagues review several sources of data which suggest an association in a small number of cases between antidepressant treatment and aggression and violence.

## Summary

Recent regulatory warnings about adverse behavioural effects of antidepressants in susceptible individuals have raised the profile of these issues with clinicians, patients, and the public. We review available clinical trial data on paroxetine and sertraline and pharmacovigilance studies of paroxetine and fluoxetine, and outline a series of medico-legal cases involving antidepressants and violence.

Both clinical trial and pharmacovigilance data point to possible links between these drugs and violent behaviours. The legal cases outlined returned a variety of verdicts that may in part have stemmed from different judicial processes. Many jurisdictions appear not to have considered the possibility that a prescription drug may induce violence.

The association of antidepressant treatment with aggression and violence reported here calls for more clinical trial and epidemiological data to be made available and for good clinical descriptions of the adverse outcomes of treatment. Legal systems are likely to continue to be faced with cases of violence associated with the use of psychotropic drugs, and it may fall to the courts to demand access to currently unavailable data. The problem is international and calls for an international response.

## Introduction

In 1989, Joseph Wesbecker shot dead eight people and injured 12 others before killing himself at his place of work in Kentucky. Wesbecker had been taking the selective serotonin reuptake inhibitor (SSRI) antidepressant fluoxetine for four weeks before these homicides, and this led to a legal action against the makers of fluoxetine, Eli Lilly [1]. The case was tried and settled in 1994, and as part of the settlement a number of pharmaceutical company documents about drug-induced activation were released into the public domain. Subsequent legal cases, some of which are outlined below, have further raised the possibility of a link between antidepressant use and violence.

The issue of treatment-related activation has since then been considered primarily in terms of possible increases in the risk of suicide among a subgroup of patients who react adversely to treatment. This possibility has led regulatory authorities to warn doctors about the risk of suicide in the early stages of treatment, at times of changing dosage, and during the withdrawal phase of treatment. Some regulators, such as the Canadian regulators, have also referred to risks of treatment-induced activation leading to both self-harm and harm to others [2]. The United States labels for all antidepressants as of August 2004 note that “anxiety, agitation, panic attacks, insomnia, irritability, hostility, aggressiveness, impulsivity, akathisia (psychomotor restlessness), hypomania, and mania have been reported in adult and pediatric patients being treated with antidepressants for major depressive disorder as well as for other indications, both psychiatric and nonpsychiatric” [3]. Despite these developments, few data are available on the links between antidepressant usage and violence. We here offer new data, review the implications of these data, and summarise a series of medico-legal cases.

This paper focuses on paroxetine primarily because we have access to more illustrative medico-legal case material for this drug than for other antidepressants. Secondly, the manufacturer, GlaxoSmithKline, submitted data on the rates of occurrence of “hostile” episodes on paroxetine for the recent review of antidepressant drugs undertaken by the British regulator [4,5]. It is not clear that the review team obtained comparable data for other antidepressants.

## Sources of Data

### Data presented to regulatory agencies

The data submitted by GlaxoSmithKline on paroxetine for review by the Committee on Safety of Medicines Expert Working Group are described as a complete set of data from all placebo-controlled trials of this drug [5]. The use of this dataset thus involves no selection by the authors, and any selection bias there might have been on the part of the company seems unlikely to have increased the size of the problem. Data from placebo-controlled trials of sertraline in children are also presented, as these also offer a complete dataset, so minimising any selection bias.

**Data from United Kingdom Drug Safety Research Unit (DSRU) prescription-event monitoring studies on paroxetine and fluoxetine [6,7].**

### Legal cases in which the authors have given evidence

We have selected these only to illustrate the range of medico-legal problems such cases can pose. In the majority of other cases in which the authors were consulted, they considered that the drug in question was not linked to the behaviour for which the defendant was charged.

**E-mails from 1,374 patients in response to a BBC programme on paroxetine broadcast in 2002.** One of us (AH) had the opportunity to analyse a complete set of these responses.

## Summary of Evidence Found

### Data from regulatory agencies

In paroxetine clinical trials, aggression and violence were commonly coded under the rubric of hostility. This coding term includes homicide, homicidal acts, and homicidal ideation as well as aggressive events and “conduct disorders”, but no homicides were reported from these trials. The material posted on the company Web site (http://www.gsk.com) suggests that these hostile behaviours in children primarily involved aggression rather than frank violence. When hostile events occurring in both adult and paediatric trials are summed, both on therapy and during the 30-day drug-free phase after taper had finished, 60 (0.65%) of 9,219 patients overall had hostile events. Table 1 shows the results [5].

**Table 1:**
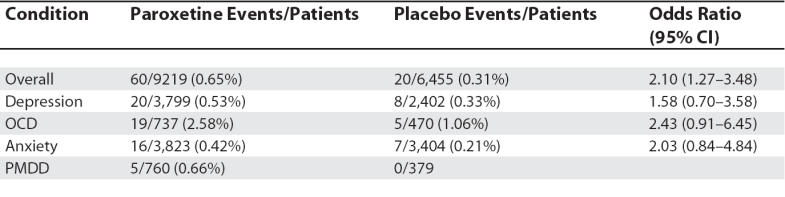
Hostility Events in Adult and Paediatric Placebo-Controlled Trials on Therapy and in Withdrawal Phase

In these trials, hostile events are found to excess in both adults and children on paroxetine compared with placebo, and are found across indications, and both on therapy and during withdrawal. The rates were highest in children with obsessive-compulsive disorder (OCD), where the odds ratio of a hostile event was 17 times greater (95% confidence interval [CI], 2.22–130.0).

In their submissions to the Committee on Safety of Medicines Expert Working Group, GlaxoSmithKline also reported that 11,491 patients entered trials comparing paroxetine with other antidepressants [5]. In this patient cohort, 44 hostile events occurred on paroxetine or other drugs, a rate of 0.38%. In the subset of trials comparing paroxetine with another SSRI, there were 16 hostile events in 2,418 patients (0.66%). These SSRI comparator trials may be confounded by indication; the SSRI comparator trials might, for instance, have included a higher proportion of patients with OCD.

Finally, in healthy volunteer studies, hostile events occurred in three of 271 (1.1%) volunteers taking paroxetine, compared with zero in 138 taking placebo [5]. Although not statistically significant, this finding is striking because hostile events are unusual in healthy volunteer trials, and this figure was higher than the rate reported in clinical populations above. GlaxoSmithKline ascribed these episodes to the fact that the volunteers were confined, although this applied to both paroxetine and placebo volunteers. One other healthy volunteer study has reported aggressive behaviour in one volunteer taking sertraline [8].

In data from sertraline paediatric trials submitted by Pfizer, aggression was the joint commonest cause for discontinuation from the two sertraline placebo-controlled trials in depressed children [9]. In these trials, eight of 189 patients randomised to sertraline discontinued for aggression, agitation, or hyperkinesis (a coding term for akathisia), compared with no dropouts for these reasons in 184 patients on placebo (95% CI, 1.72–infinity). When discontinuations for any manifestation of treatment-induced activation (suicidal ideation or attempts, aggression, agitation, hyperkinesis, or aggravated depression) were considered, there were 15 discontinuations on sertraline compared with two on placebo, a relative risk of 7.3 (95% CI, 1.70–31.5; *p* = 0.0015). The report of these studies does not include an analysis of these data [9]. In the only other placebo-controlled sertraline paediatric trial, undertaken in children and adolescents with OCD, there were ten dropouts out of 92 patients on sertraline, five of whom discontinued for behavioural activation, two for agitation, one for aggression, one for nervousness, and one for emotional lability. In comparison, there was one discontinuation for hyperkinesis out of a total of two dropouts from 95 patients on placebo [10].

Finally, in paediatric trials of venlafaxine (Wyeth), two percent of children dropped out because of hostility, more than double the rate of dropout on placebo [11].

By 2003, 121 cases of aggression on paroxetine had been reported to the Medicines and Healthcare Products Regulatory Agency (MHRA), and by January 2006 that number had risen to 211 [12]. It should be noted that such reporting systems estimate that physicians report between one and ten percent of adverse effects on treatment [13].

### DSRU data

Evidence from two DSRU prescription-event monitoring studies of paroxetine and fluoxetine [6,7] is shown in Table 2, summarising details of aggressive events and assaults in patients prescribed fluoxetine and paroxetine in primary care after the launches of these two drugs. These data are consistent with the clinical trial data reported above. The greatest frequency of events was during the first month of treatment (unpublished data).

**Table 2:**
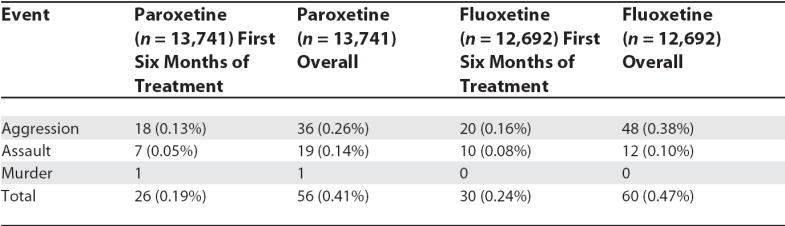
DSRU: Prescription-Event Monitoring Studies of Paroxetine and Fluoxetine

### The medico-legal cases

Nine illustrative cases in which we have between us acted as expert witnesses are summarised in Table 3. In eight of them the person who was taking an antidepressant was the defendant; in one (DS; see Annex), the patient killed three members of his family and then himself, and his son-in-law sued SmithKline Beecham. We have chosen the cases to demonstrate the diversity of the issues they raise. They are described in the Annex.

**Table 3:**
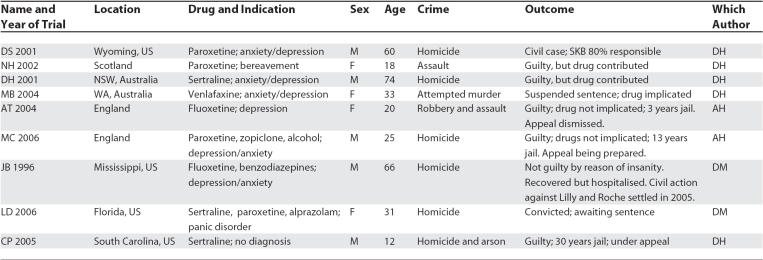
Summary of Illustrative Cases

### E-mails from patients to a BBC television programme

After a programme on paroxetine in 2002, the producers of the BBC television programme *Panorama* received 1,374 e-mails from viewers, mostly patients. One of us (AH) was able to analyse the full set of these responses. Many linked emotional storms and thoughts and acts of violence or self-harm to paroxetine, both to starting drug treatment and to dosage change. These were not simple anecdotal reports, in that the analysis clearly pointed to a linkage with dosage. Second, they were self-reports of violence from patients with no apparent background of violent behaviour [14]. Third, the analysis was consistent with an analysis of reports of thoughts and acts of violence or self-harm on paroxetine that doctors had sent to the MHRA about other patients between 1991 and 2002 [15]. In both patient and medical reports, severe mood changes were commonly associated with changes of drug dosage during the first week of treatment, with later dosage increase, or with dosage decrease or drug withdrawal. The accounts reported in both the medical and the patient series had much in common, including time frame and a linkage to dosage [15].

## Discussion

### Mechanisms of antidepressant-induced violence

A link between antidepressant use and violence needs a plausible clinical mechanism through which such effects might be realised. There are comparable data on increased rates of suicidal events on active treatment compared to placebo [16,17]. In the case of suicide, several explanations have been offered for the linkage. It is argued that alleviating the motor retardation of depression, the condition being treated, might enable suicides to happen, but this cannot explain the appearance of suicidality in healthy volunteers. Mechanisms linking antidepressant treatment, rather than the condition, to adverse behavioural outcomes include akathisia, emotional disinhibition, emotional blunting, and manic or psychotic reactions to treatment. There is good evidence that antidepressant treatment can induce problems such as these and a prima facie case that akathisia, emotional blunting, and manic or psychotic reactions might lead to violence.

### Akathisia

Some of the best descriptions of akathisia come from the medical literature on the use of reserpine as an anti-hypertensive in the mid-1950s [18]:

“Increased tenseness, restlessness, insomnia and a feeling of being very uncomfortable”.

“On the first day of treatment he reacted with marked anxiety and weepiness, on the second day felt so terrible with such marked panic at night that the medication was cancelled”.

“The first few doses frequently made them anxious and apprehensive. They reported increased feelings of strangeness, verbalised by statements such as ‘I don't feel myself’ or ‘I'm afraid of some of the unusual impulses I have'’.

Events such as these in clinical trials of antidepressants have commonly been coded under headings such as agitation, emotional lability, and hyperkinesis (overactivity), and only rarely to akathisia. In clinical practice the term has sometimes been restricted to states of demonstrable motor restlessness, but by definition it cannot be a simple motor disorder or it would be classified as a dyskinesia [19]. There is good evidence that akathisia can exacerbate psychopathology in general [20] and consensus that it can be linked to both suicide and violence [21,22]. A link between akathisia and violence, including homicide, following antipsychotic use has previously been reported [23–25].

Substantial evidence from SSRI clinical trials shows that these drugs can trigger agitation. Approximately five percent of patients on SSRIs in randomised trials drop out for agitation against 0.5% on placebo. The current data sheets for SSRI antidepressants specify that the drugs can cause akathisia and agitation, and warn about developing suicidality in the early phase of treatment, on treatment discontinuation, and in the wake of a dosage increase during the course of treatment. In the US, these warnings explicitly apply to not only depressed patients but also people being treated for anxiety, smoking cessation, or premenstrual dysphoric disorder. In Canada, warnings specify an increased risk of violence in addition to suicide.

### Emotional blunting

Another mechanism that may contribute to hostile events is treatment-induced emotional blunting. Several reports published since 1990 have linked SSRI intake with the production of emotional blunting, detachment, or an amotivational syndrome, described in one report as the equivalent to a “chemical lobotomy” [26–29]. It is quite common in clinical practice to find people who say they simply are not bothered any more. Things that would previously have worried them no longer do so. However, clinical trials of antidepressants have so far not assessed this phenomenon and its frequency is not reliably known.

### Mania and psychosis

Another mechanism that may link SSRIs to violence are the manic or psychotic states reported to be induced by drug treatment. These drug-induced states often resolve once the medication is removed. However, the full dimensions of treatment-induced psychotic or manic reactions have yet to be mapped; some may continue for a long period after treatment has stopped [30]. It has recently been estimated that these drug-induced manic or psychotic states may account for up to eight percent of admissions to psychiatric facilities [31–35].

The development of a psychotic episode or of command hallucinations has traditionally been linked to both violence and suicide. The labels for most SSRIs now concede a causal relationship to psychosis and to hallucinations.

A proportion of these cases with superficially manic or psychotic reactions and unrecognised confusion may be delirious states reflecting organic brain disturbances rather than a functional psychosis or mania. Delirium is an absolute defence against murder, while psychosis and mania may not be.

### Somnambulism

Another mechanism that may be relevant to violence and murder is sleepwalking. Somnambulism can provide an absolute defence against murder, in that the defendant in such a case does not have the capacity to form intent. Several reports have been published of an association of paroxetine with sleepwalking in people not previously known to have sleepwalked [36,37]; somnambulism has also been reported for other SSRIs [37]. Among the drugs linked to sleepwalking in reports to the UK MHRA up to January 2006, paroxetine came second with 12 reports, and zopiclone first with 13 reports, with antidepressants occupying eight of the top 17 slots.

Paroxetine has also been reported to the MHRA more often than any other drug for nightmares (206 reports). The second most commonly reported drug is mefloquine (Lariam), a drug noted for triggering psychosis, with 132 reports. Antidepressants occupy six of the top ten slots for reports of nightmares. As mentioned above, clinicians report between one and ten percent of adverse events to regulators and thus the incidence of nightmares on paroxetine is substantial.

## What Our Findings Add to Earlier Reports

Our main finding is that unselected sets of placebo-controlled trials of antidepressants show evidence for an increased relative risk of aggressive behaviours on treatment, although such outcomes apply to only a small subset of patients. The relative risks cited here reflect a net balance of treatment-induced benefits and adverse outcomes. If treatment with an antidepressant, such as paroxetine, lowers the overall risk of aggression in a proportion of patients in a trial population, then the real rate of treatment-induced difficulties with paroxetine may be somewhat higher than the net figures from placebo-controlled trials indicate. Studies in healthy volunteer populations in which treatment would not be expected to reduce aggressive episodes stemming from an underlying clinical condition might help clarify this point.

Data from pharmacovigilance studies support these clinical trial findings, and the literature on antidepressant drugs offers several plausible mechanisms through which such effects might be mediated.

One strength of the current study is that the data are unselected. The data are consistent, although they come from a variety of sources. A weakness of the study is that we have been able to include only a subset of existing data in the analysis. Data on aggression on other antidepressants will necessarily have been collected as part of the development programmes for these drugs, but these data are not in the public domain. The sample of patients cited here is therefore relatively small, especially when selected age-groups and indications are considered. The wide confidence intervals reflect these limitations.

Earlier reports have linked antidepressants to violence [38], but this is the first independent study to offer a quantitative analysis of the issue; no other studies exist with which our results can be compared.

## Legal Implications

The legal system has in recent years been faced with a number of cases of violence in which antidepressant treatment may have played a part. If antidepressants can in principle trigger violence, a need will always remain to establish whether such a general possibility might have been realised in an individual case. The principles involved in making such assessments will involve a consideration of the timing of the events in relation to treatment, the merits of competing explanations, and the existence of evidence in a particular case for a mechanism through which treatment may have led to violence.

At present, different jurisdictions take differing approaches to the issue of whether treatment with a prescription drug can be invoked as a possible defence or mitigating factor in cases of murder or violence. The question of what legal defences are appropriate in such cases needs to be addressed, as do the possible implications of such defences for a defendant and society.

Broadly speaking, treatment-related difficulties of this sort fall under the heading of automatisms. An automatism is defined as a transient, non-recurrent mental malfunction caused by an external factor, whether physical or psychological, that the mind of an ordinary person would be unlikely to have withstood and that produces an incapacity to control his or her acts. However, the question of automatisms has not been mapped onto the domain of potential problems that might result from prescription drug use, as outlined here.

In the DS and DH cases (see Annex), it seems reasonable to argue for an automatism. These men may have been overwhelmed by the effects of prescribed medication to the extent that they may not have been able at the time to form a clear intention to engage in the acts that resulted in the deaths of their families. The case of MC may have involved a case of sleepwalking, which provides a classic defence of automatism. The CP case may have involved command hallucinations. JB had a clear delusional belief system and was therefore found not guilty by reason of insanity.

If these cases are relatively straightforward medico-legally, the cases of NH, MB, AT, and LD are more complex, and may require medico-legal developments. The notion of an automatism is typically invoked to cover behaviours occurring during events such as sleepwalking or epileptic seizures, where normal consciousness is significantly disturbed and the disturbance is of acute onset and brief duration. In contrast, MB, NH, and LD found themselves involved in an extended disturbance, in which consciousness was functioning well enough to allow them to maintain the semblances of normal behaviour for several weeks. Aside from the element of duration, there is a further factor. The situation is more like that of someone whose drink has been adulterated. In such circumstances, some of those affected may guess what has happened and be able to compensate for the hazard, while others may not. In the case of these prescription drugs, one of the mechanisms by which an individual might compensate is to check with his or her physician. In the cases of NH and LD, perceptions of difficulties may have been confounded by professional advice that the drug could not be the source of the problem.

If an element of the hazard posed by treatment stems from a lack of warnings or information, one might argue a particular case against the background of current or recent warnings. Should these drugs in due course come complete with clear warnings that were implemented in clinical practice, one might potentially take a quite different view, closer to the view taken about alcohol and violence.

Further complexities emerge in considering some of the mechanisms listed above. For instance, in the case of AT, how should the possibility of emotional blunting be handled? In the case of a drug that quells normal fearful responses and concern for consequences, it is difficult to know how to determine degrees of responsibility.

For this area to move forward, more data are needed. Pertinent clinical trial data have been generated but remain unavailable. Combining datasets might make it possible to establish whether the risks of treatment are related to age and gender, or whether those with and without prior histories of aggression are affected similarly. While it may be that further data would show that the risk associated with certain SSRIs and tricyclic antidepressants may be less than others, or may not exist in all antidepressants, there is no way to make that determination without access to these data. Indeed, the issue of violence triggered by older antidepressants has been raised before [38]. Current warnings in the US and Canada are consistent across antidepressants, but in other countries, for instance in the UK (see Summaries of Product Characteristics on the Electronic Medicines Compendium Web site, http://emc.medicines.org.uk), the wording differs from drug to drug. Given the new medico-legal issues some of these cases pose, it may well fall to the courts to demand that data now unavailable be made public.

## Conclusion

The new issues highlighted by these cases need urgent examination jointly by jurists and psychiatrists in all countries where antidepressants are widely used. The problem is international, and it would make sense to organise an international effort now.

In practice, clinicians need to be aware of the issues, but serious violence on antidepressants is likely to be very rare. When violence is a suspected outcome, every case has to be considered carefully, on the principle that individuals are responsible for their conduct, unless there is clear evidence of compromised function that cannot be otherwise explained.

## Annex: The Illustrative Medico-Legal Cases

### Case 1

DS was a 60-year-old man with a history of five prior anxiety/depressive episodes. These did not involve suicidality, aggressive behaviour, or other serious disturbance. All prior episodes had resolved within several weeks. In 1990 DS had had an episode of depression, which his doctor treated with fluoxetine. He had a clear adverse reaction to fluoxetine involving agitation, restlessness and possible hallucinations, which worsened over a three-week period despite treatment with trazodone and propranolol that might have been expected to minimise the severity of such a reaction. After fluoxetine was discontinued DS responded rapidly to imipramine.

In 1998, a new family doctor, unaware of this adverse reaction to fluoxetine, prescribed paroxetine 20 mg to DS, for what was diagnosed as an anxiety disorder. Two days later having had, it is believed, two doses of medication, DS using a gun put three bullets each through the heads of his wife, his daughter who was visiting, and his nine-month-old granddaughter before killing himself.

At jury trial in Wyoming in June 2001, instigated by DS' surviving son-in-law, a jury found that paroxetine “can cause some people to become homicidal and/or suicidal” [39]. SmithKline Beecham was deemed 80 percent responsible for the ensuing events [1]. The documentary evidence included an unpublished company study of incidents of serious aggression in 80 patients, 25 of which involved homicide.

Experts for the plaintiff suggested that the mechanism through which paroxetine contributed to these events was probably akathisia or psychosis. A central problem with both akathisia and psychosis in such contexts is that the takers of medications often fail to recognise the fact that the state they are in is drug-induced and that discontinuing treatment can alleviate the symptoms.

### Case 2

NH was 18 when prescribed paroxetine 20 mg/day by her general practitioner (GP) in Scotland following the death of her grandmother, at the end of November 2001. Within days, she became markedly somnolent, agitated, and emotionally labile. There was an increasing series of arguments at home, and unprecedented aggression. After eight weeks, her parents, concerned about the situation, brought her back to the GP, who increased the dose of paroxetine to 30 mg. One week after the increase of dose and two months after the initial prescription, NH was involved in an incident at a nightclub in which she assaulted another person.

The dose of paroxetine was reduced to 20 mg. Her behaviour remained unstable, disinhibited, and there was at least one suicidal act. Three months later she stopped treatment. She had significant withdrawal problems, but her behaviour normalised. Having been out of work for close to a year she went back to work and has remained in employment since.

NH pled not guilty by virtue of an automatism. The case was heard in open court where the jury found her guilty but added “that antidepressants had contributed to her actions on the day in question”. The judge imposed a suspended sentence, stating that “but for Seroxat you wouldn't be standing here”. This case appears to have involved treatment-induced akathisia.

### Case 3

DH was a 74-year-old man from New South Wales with a history of mixed anxiety/depressive episodes, many of which resolved without drug treatment. He had no history of violence or suicidality, and had remained gainfully employed throughout.

During one of these episodes, DH was given sertraline (Zoloft) by a GP and clearly responded adversely to this, most notably with agitation. He stopped treatment the following day on medical advice. In July 1999, he sought help from his GP, who was on leave. DH was seen by a locum who admitted in Court that he had not checked DH's file before prescribing sertraline 50 mg. That night, apparently feeling worse after a first dose of sertraline, DH took four more doses of sertraline.

The next morning, after his wife got up he met her in the kitchen and strangled her. He then set off in his car, having decided to kill himself, but turned round and contacted the police to tell them what had happened. He decided he should accept the consequences of his actions and did not want to distress his family further.

DH's lawyers had intended to defend the case on the basis of non-insane automatism or involuntary intoxication, but before the proceedings in May 2001, the Crown made an offer that if DH pleaded guilty to manslaughter on the basis of substantial impairment, the Crown Prosecutor would not contest any defence submission that DH be released from gaol on the date of his sentence. Further, the Crown accepted the case put forward by the defence implicating sertraline. DH accepted that offer in view of his age (78). The judge in his summing-up released DH and stated: “I am satisfied that but for the Zoloft he had taken he would not have strangled his wife” [40].

This case might best be explained in terms of a treatment-induced akathisia or delirious state.

### Case 4

MB was a 33-year-old woman with two children who had untreated nervous problems since her teenage years. In 2001 she approached her GP who prescribed paroxetine. An initial 20 mg dose was increased to 30 mg. MB appeared to become more anxious and agitated. This deterioration led to a switch to venlafaxine, which was successively increased to 300 mg/day. During these increases, the medical notes record her as being more anxious and agitated, but did not link this to treatment.

She made plans to take her own and her children's lives, and taking the children for a drive, attached a hosepipe to the exhaust. In the course of two efforts to execute this plan, she thought better of it and informed both the police and child-care authorities what had happened. Her children were taken into care and she was charged with attempted murder.

During the sentencing in the Supreme Court of Western Australia in April 2004, the judge stated there were substantial grounds for implicating venlafaxine in MB's behaviour, and gave her a suspended sentence [41]. This case again appears to involve treatment-induced akathisia.

### Case 5

AT had a baby daughter in December 2000 at the age of 17. In June 2003, her GP noted that she had been “low for 2 years, worse recently”, and prescribed fluoxetine 20 mg. Before treatment she was noted to be “self-harming with superficial abrasions to her lower limbs underneath her trousers and has been thinking of hanging herself. She has not planned to as she would not do that to her daughter and has no immediate plans of suicide of any description.”

Three weeks later she robbed a 14-year-old boy of his phone and watch. Two days later she stole another phone. Four days later, a psychiatrist noted: “She tells me that the intensity and the distress caused by [the suicidal] thoughts have subsided since starting treatment with fluoxetine. [She] feels that her mood did initially improve on fluoxetine but that this effect is now wearing off.” He concluded “it seems that she has partially responded to treatment with fluoxetine…I have advised her to increase the dose of fluoxetine to 30mg in the morning.” She did as advised but the day after, as well as five days later, she engaged in further robberies. Three weeks later she attempted robbery with an offensive weapon.

In October, a forensic psychiatrist examining her in prison noted that for the preceding two months, while in prison she had been prescribed mirtazapine 30 mg nightly (a non-SSRI), and had become calmer and better able to discuss her situation. The writer “would now be surprised if she reverted to her [previous criminal] behaviour.”

AT had never before been involved in criminal behaviour. Her first two offences took place 17 and 19 days after she started fluoxetine. They appear to have been impulsive and were marked by complete lack of feeling. The third, fourth, and fifth offences occurred after a dose increase. The fifth offence involved brutal violence and use of a flick knife. The prison assessment took place when she had been off the drug for about ten weeks, long enough to eliminate the drug.

Her final charges involved robbery and assault as well as child neglect. Based on the medical records, one of us (AH) noted in his report to the court that AT appeared to have suffered treatment-induced emotional blunting. However, the judge in this English case doubted that the effects of the drug could explain the deliberate planning of robberies and she was found guilty and sentenced to three years in prison with no allowance for any contribution from fluoxetine. An appeal was rejected.

### Case 6

MC started drinking alcohol socially in 1995 at the age of 17. He used ecstasy in 1999 but stopped after a bad experience. He began using cocaine from February 2001, increasing during October through to June 2002, ultimately using 6 g/day for a short time. After July 2002 MC's cocaine use reduced to nil, apart from four minor relapses. He had none after May 2003. MC's alcohol use increased to four to five cans of lager a night in 2002.

He was prescribed paroxetine 20 mg/day for depression in late May 2002. During the first two months on paroxetine he experienced “terrible shaking of the hands; couldn't pick up a glass of milk without spilling it”, felt nausea and had “a constant dull headache, as if squinting in sunlight”. When he missed a tablet of paroxetine, he wanted to hide under a duvet and to stay away from everybody; his hands shook, and he had headaches and nausea. These symptoms lasted a couple of days, and he learned not to miss a dose.

In September, his GP increased paroxetine to 30 mg “because he was still very anxious”, and advised him to take the paroxetine earlier, when its stimulant effects would be more acceptable, rather than late. He was also started on a regular zopiclone prescription at this point to counter paroxetine stimulation. Soon after, another doctor in the practice changed him to the more sedating dothiepin, but after a few weeks he asked to be put back on paroxetine. He subsequently stopped cocaine but began drinking more heavily. Prescriptions of paroxetine and zopiclone continued through to July 2003.

At this stage he was estranged from an ex-partner with whom he had a now 18-month-old daughter. In August 2003, at her home, after ten pints of lager, he took two zopiclone tablets. Following an argument, they had a pint of beer each, during which there was another bout of quarrelling, and she went to bed alone, leaving him to sleep on the sofa. MC may have taken four more zopiclone tablets. He appeared later that night blood-stained in the local police station with his daughter in his arms. The police found his partner dead from multiple stab wounds. He was charged with murder.

In prison paroxetine 30 mg was continued; zopiclone was stopped. During his initial period on paroxetine, and then in prison, MC complained of “terrible nightmares, waking dripping with sweat, soaking the bed”. Intense frightening nightmares have been reported regularly in healthy volunteers taking paroxetine. MC had no reported episodes of sleepwalking before using paroxetine, but he had a number of documented episodes of sleepwalking after starting the drug, and two first-degree relatives had a history of sleepwalking. Sleepwalking has been reported in association with zolpidem, a hypnotic related to zopiclone [42–44], but no case of sleepwalking on zopiclone has been reported in the scientific literature. However, as noted above, zopiclone is the drug most commonly linked to sleepwalking in Yellow Card reports to the MHRA.

Clearly violence follows domestic arguments, and is a known effect of alcohol, but this case offers grounds also to implicate paroxetine and zopiclone. Zopiclone is known to cause a dose-dependent confusion and amnesia comparable to that found with benzodiazepines [45]. Violence cannot however be attributed to a direct effect of paroxetine alone, since MC had been maintained on this for almost one year with no prior violence. In these circumstances MC pleaded guilty at his trial on 27 February 2006. The judge did not accept that paroxetine and zopiclone had played any part, and sentenced him to 13 years prison. An appeal against the sentence is being prepared.

### Case 7

JB was 66 years old, married to a second wife ten years his junior. They had marital difficulties, with frequent arguments but no history of violence. JB had medical complaints and longstanding depression and anxiety. Digestive symptoms were treated with an antispasmodic combined with chlordiazepoxide (5 mg four times daily); generalised anxiety was also treated with chlordiazepoxide (10 mg twice daily); an undiagnosed movement disorder, characterized by twitches and tics, was treated with clonazepam (0.5 mg at night). In addition, JB had been treated with the antidepressant doxepin 75 mg at night for years.

Concerned about the sedative effects of his medication, JB's wife began replacing active doxepin powder with sugar in an attempt to offset this effect. JB suspected the capsules had been tampered with. His wife admitted doing this when they saw a new psychiatrist in mid-August 1994. The doctor considered JB to have major depression with anxiety, complicated by physical symptoms and marital strife. He noted that JB was not psychotic or suicidal, and agreed that doxepin be discontinued, instead prescribing fluoxetine 10 mg daily, continuing the other medications as before.

JB was meticulous about compliance and even kept a medicines log. He remained concerned that his wife was tampering with his pills, and after four weeks fluoxetine accused her of being unfaithful. Alarmed at his suspicions, his wife rang the psychiatrist and disposed of the household gun. Meanwhile, JB's friends noted that, normally placid, he had become tense, strange, and suspicious; he asked for a replacement gun to defend himself; described a plan to escape an expected attempt on his life; feared poisoning of food and drink; feared an ambush when visiting his mother's grave. Two months after starting fluoxetine JB had become floridly deluded, expecting to be attacked or poisoned by his wife, or her agent. The psychiatrist received phone calls of concern from friends and family but did not alter his treatment. One evening in mid October 1994 JB approached his neighbours, covered in blood, reporting an attack by his wife. He had several minor cuts to his arms. His wife was found dead in their hallway, in a pool of blood with 200 stab wounds.

In 1996, a Mississippi court found JB not guilty of murder by reason of insanity [46]. He was confined to a mental hospital, where he remains, even though on review of his medical notes by one of us (DM), it was clear that his psychosis cleared on withdrawal of fluoxetine, and further treatment. His physicians are concerned about the risk should he be discharged. Although prescription drugs were not invoked in his defence, a subsequent civil case seeking damages from Eli Lilly (Prozac) and Hoffman LaRoche (benzodiazepines) was settled in 2005 (personal communication from plaintiff's lawyer, R. Boyd). This homicide case involves a treatment-induced psychosis.

### Case 8

LD, a 31-year-old mother, separated from the father of her 3-year-old twin boys in 2001. After a protracted custody battle, she began experiencing episodes of dizziness, sweating, shaking, nausea, and pressure in the chest. She was well between episodes, experienced no suicidality, irritability, or aggression, and continued to care for her sons as before, living in the same house as her father and his second wife.

Reading a magazine, she saw an advertisement for “panic disorder”, and recognised many of the symptoms described in it. She contacted her family doctor, but no appointments were available and she saw the nurse practitioner instead. She was given a free starter pack of sertraline 25 mg, and a prescription for alprazolam 0.5 mg twice daily to start immediately.

LD found the drugs stopped her panic attacks, but she experienced increasing tension, restlessness, and agitation, which worsened when the “starter pack” dose of sertraline increased after one week from 25 to 50 mg/day. Other unexpected effects were that her previous moderate alcohol intake took on a compulsive quality, and she became increasingly depressed and began to think of suicide. On one occasion she found herself in the closet holding her father's pistol before “coming to” and realising what she was doing. Alarmed, she tried to see her doctor, but he was not available. She again saw the nurse, who switched her from sertraline, which she had taken for a month, to a starter pack of paroxetine 20 mg/day and advised continuing alprazolam at 1 mg/day.

LD's agitation, restlessness, depression, and suicidal ideas worsened. Two days after the switch to paroxetine, she claims she took double the prescribed amount of both paroxetine and alprazolam, hoping this would help. It didn't. She drank alcohol and sounded intoxicated on the phone. Claiming she saw no future for herself or her children, she shot both in the head just before their afternoon nap. She recalls intending to kill herself as well, but did not do this immediately as she noticed one son was still breathing. Unwilling to “leave him behind”, she waited but passed out from her overdose of alprazolam and alcohol, and was discovered deeply asleep with her twins dead next to her. Her blood and urine alcohol levels showed marked intoxication.

The Florida State Attorneys initially sought to have LD convicted of murder and sentenced to death, but later dropped pursuit of the death penalty. The defence team contended that LD was not guilty by reason of temporary insanity caused by the prescription drugs provided by the nurse practitioner. Prior to trial a “Frye” hearing was held to consider whether evidence regarding SSRI-induced akathisia, involuntary alcohol intoxication, suicidality, and homicidality would be admissible. The judge ruled that evidence could be admitted indicating that akathisia was *associated* with SSRI treatment, but that a *causal* relationship could not be argued. With this restriction on defence testimony, the State Attorneys convinced the jury that the drugs did not play a causal role in the homicides. LD was convicted, and sentenced to life without possibility of release [47].

### Case 9

According to an independent forensic report compiled a year after the events for which CP was charged in November 2001, CP was a 12-year-old, 5'2”, 95-lb boy with a family background involving considerable social dislocation. Despite the difficulties of his social situation, he had no record of treatment for nervous disorders or of violence or behavioural disturbance. Following an argument with his father at the end of October 2001, he was admitted to a behavioural centre for six days where he was started on paroxetine. His behaviour worsened daily on paroxetine. He was discharged against medical advice to the care of his grandparents, who, when his paroxetine ran out, took him to their primary-care physician who prescribed sertraline 50 mg, increasing this to 100 mg two days before the killings for which CP was charged. The duration of sertraline treatment was three weeks.

After the prescription of sertraline, CP was involved in a number of aggressive incidents at school, the first on record for him, and was reported by family members and church members to be restless and talking unusually volubly. Relatives noted a series of risky behaviours. On the day of the killings, his grandparents had told him that he could not take the school bus following an episode of aggression toward one of the other children on the bus. Later that evening he attended choir practice with his grandparents, who in response to escalating difficulties had warned him he might have to be returned to his father.

The independent forensic report on the case notes CP as saying that that night: “something told me to shoot them”. He had initially reported this to be hallucinations and then said he thought it was his own thoughts. When asked to specifically describe what the experience was like, he said it was “like echoes in my head saying ‘kill, kill’, like someone shouting in a cave”. According to the forensic report, “He reported this began happening after he went to bed…He reported he had never considered harming his grandparents before and this was unlike anything he had previously experienced. He reported that the voices were coming from inside his head and they bothered him so much that he got up. He reported that the voices continued until he killed his grandparents. He reported that he couldn't control himself and reported the echoes stopped after he shot his grandparents. He set fire to the house but could not explain these actions saying the thoughts just popped up”. He then took a vehicle and began driving but reported that he had no idea where he was going and that it all felt like a dream. He recalled asking the police about his grandparents after he was picked up because he was not sure if it had really happened or not.

These events and CP's overall behaviour and history led an independent forensic child psychiatrist to diagnose substance-induced mania and psychotic disorder. The charges of double murder and arson were heard by jury trial in an adult rather than juvenile court. In the process of jury selection, 32 of 75 prospective jurors declared that they or someone related to them were on or had been on an antidepressant. Court TV covered the trial in its entirety. Both prosecution and defence from the outset accepted that CP had shot his grandparents. Media coverage focused heavily on the question of “‘Evil’ or ‘chemically compelled’?”

In February 2005, after a two-week trial, a jury found CP guilty of murder and he was sentenced to 30 years in prison [48]. Questioned by the media afterwards, “Steven Platt, a 26-year-old accounting clerk for an electrical supply wholesaler, said the group believed that Christopher exhibited side effects from Zoloft but did not feel it was severe enough to let him escape criminal responsibility” [49]. Summing up some of the points at issue, the judge Daniel Pieper stated: “There is no case in South Carolina that addresses involuntary intoxication by prescription drugs…It seems to turn the whole medical system on its side if you can't rely on the medication your doctor prescribes. It could potentially force you into a situation of lifetime commitment if that drug induces an effect of which you're not aware… There's something disconcerting about that, albeit probably something of a legal nature that is troubling me” [50]. The verdict is currently under appeal in the South Carolina Supreme Court.

## Abbreviations

CI: confidence interval
DSRU: Drug Safety Research Unit
GP: general practitioner
MHRA: Medicines and Healthcare Products Regulatory Agency
OCD: obsessive-compulsive disorder
SSRI: selective serotonin reuptake inhibitor
